# The impact of late‐onset epilepsy as a co‐occurring condition of Alzheimer’s disease on tau levels in older adults with Down syndrome

**DOI:** 10.1002/alz.092652

**Published:** 2025-01-09

**Authors:** Nazek Queder, Liv McMillan, Mithra Sathishkumar, Lisa M. Taylor, David B. Keator, Eric Doran, Christy L. Hom, Sharon J. Krinsky‐McHale, Melissa Petersen, Sid E. O'Bryant, Adam M. Brickman, Elizabeth Head, Mark Mapstone, Wayne Silverman, Ira T. Lott, Michael A. Yassa

**Affiliations:** ^1^ University of California, Irvine, Irvine, CA USA; ^2^ New York State Institute for Basic Research in Developmental Disabilities, Staten Island, NY USA; ^3^ University of North Texas Health Science Center, Fort Worth, TX USA; ^4^ Columbia University, New York, NY USA

## Abstract

**Background:**

There is an increased risk of epilepsy in Down syndrome (DS), especially after the age of 40. The onset of this co‐occurring disease is closely associated with Alzheimer’s disease (AD) related cognitive deterioration. Therefore, understanding the impact of late‐onset epilepsy on AD biomarkers in people with DS is crucial. Here, we aim to test the difference in tau levels and hippocampal volume in people with late‐onset seizures, childhood‐onset seizures, and no seizures.

**Method:**

We analyzed data from 54 participants (52 +/‐ 6.5 years, 66% females) with DS enrolled in the Alzheimer’s disease Down syndrome study. A total of 18 participants had a history of seizures: 9 participants had recent onset of seizures in the last 5 years and 9 participants had childhood‐onset of seizures. A 2‐to‐1 match method was used to identify 36 control match participants based on age, sex, and dementia status. Magnetic resonance imaging (MRI) and flortaucipir (FTP) positron emission tomography (PET) were used to assess atrophy and tau accumulation respectively. Plasma ptau181 levels were assessed using the ultra‐sensitive single molecule array (Simoa) technology platform HDX imager. ANOVA was used to assess group differences.

**Result:**

The results showed significant differences in ptau181 levels between individuals with late‐onset seizure disorder, individuals with childhood‐onset of seizures, and controls (F(2,51)= 9.649; *p*<.001). Post‐hoc analysis revealed that individuals with late‐onset seizure disorder had significantly higher levels of ptau181 compared to individuals with childhood‐onset (*p*<.001) and controls (*p* = 0.002), with no differences between childhood‐onset seizures and controls. No differences were observed in the hippocampus volume among the three groups. Differences in the temporal meta‐ROI tau SUVR were assessed in a subset of our sample. We observed a trend in individuals with history of seizures showing higher tau SUVR in the temporal meta‐ROI compared to controls.

**Conclusion:**

Our findings suggest that the levels of ptau181 could be impacted by late‐onset seizure disorder. These results shed the light on the importance of assessing the impact of co‐occurring disorder on AD related biomarkers. Future research will explore the impact of late‐onset seizure disorder on AD‐related brain biomarkers longitudinally.

